# Thymic B Cells Promote Germinal Center-Like Structures and the Expansion of Follicular Helper T Cells in Lupus-Prone Mice

**DOI:** 10.3389/fimmu.2020.00696

**Published:** 2020-04-28

**Authors:** Yessia Hidalgo, Sarah Núñez, Maria Jose Fuenzalida, Felipe Flores-Santibáñez, Pablo J. Sáez, Jessica Dorner, Ana-Maria Lennon-Dumenil, Victor Martínez, Emmanuel Zorn, Mario Rosemblatt, Daniela Sauma, Maria Rosa Bono

**Affiliations:** ^1^Departamento de Biologia, Facultad de Ciencias, Universidad de Chile, Santiago, Chile; ^2^Cells for Cells-Consorcio Regenero, Facultad de Medicina, Universidad de los Andes, Santiago, Chile; ^3^Fundacion Ciencia & Vida, Santiago, Chile; ^4^INSERM U932, Institut Curie, Centre de Recherche, PSL Research University, Paris, France; ^5^FAVET-INBIOGEN, Faculty of Veterinary Sciences, University of Chile, Santiago, Chile; ^6^Department of Medicine, Columbia Center for Translational Immunology, Columbia University Medical Center, New York, NY, United States; ^7^Facultad de Ciencias de la Vida, Universidad Andres Bello, Santiago, Chile

**Keywords:** systemic lupus erythematosus, thymic B cells, germinal center, plasma cells, follicular helper T cells

## Abstract

Systemic lupus erythematosus (SLE) is an autoimmune disease characterized by the activation of autoreactive T and B cells, autoantibody production, and immune complex deposition in various organs. Previous evidence showed abnormal accumulation of B cells in the thymus of lupus-prone mice, but the role of this population in the progression of the disease remains mostly undefined. Here we analyzed the spatial distribution, function, and properties of this thymic B cell population in the BWF1 murine model of SLE. We found that in diseased animals, thymic B cells proliferate, and cluster in structures that resemble ectopic germinal centers. Moreover, we detected antibody-secreting cells in the thymus of diseased-BWF1 mice that produce anti-dsDNA IgG autoantibodies. We also found that thymic B cells from diseased-BWF1 mice induced the differentiation of thymocytes to follicular helper T cells (T_FH_). These data suggest that the accumulation of B cells in the thymus of BWF1 mice results in the formation of germinal center-like structures and the expansion of a T_FH_ population, which may, in turn, activate and differentiate B cells into autoreactive plasma cells. Therefore, the thymus emerges as an important niche that supports the maintenance of the pathogenic humoral response in the development of murine SLE.

## Introduction

Systemic lupus erythematosus (SLE) is an autoimmune disease characterized by a set of clinical abnormalities ranging from mild symptoms such as malaise, arthritis, or dermatitis to more severe manifestations such as renal disease or compromise of the central nervous system. At the immunological level, SLE patients exhibit hyperactivity of T and B cells against self-antigens that leads to the secretion of autoantibodies against nuclear components such as DNA, RNA, histones, ribonucleoproteins, among others. Autoantibodies bind antigens and form immune complexes that deposit in the skin, joints, kidneys, heart, and central nervous system, generating inflammation and damage (1, 2).

The thymus is a primary lymphoid organ whose main function is the induction of immune self-tolerance to prevent autoimmunity. This organ is dedicated to T cell generation and maturation, a function that usually declines with age and can be severely compromised in autoimmune diseases (3). It has been described that patients with autoimmune conditions such as myasthenia gravis exhibit alterations in the thymic structure and its cellular components (4–6). One of the important alterations in the thymus of patients with myasthenia gravis is the increase of autoreactive B cells. In the literature, there have been reports of patients with other autoimmune diseases, such as ulcerative colitis and systemic lupus erythematosus that present abnormalities in the structure of the thymus (7–9). However, it is unknown if these changes contribute to the disease.

B cells are a scarce population in the thymic medulla of both, healthy humans and mice (~0.3% of total cells) where they are thought to function as antigen-presenting cells for thymic selection (10–13). We have previously shown that throughout normal aging, the human thymus accumulates at perivascular spaces memory B cells and plasma cells that generate antibodies with antiviral reactivity (14). Remarkably, a subset of myasthenia gravis patients has been diagnosed with thymic follicular hyperplasia which encompasses a considerable expansion of B cells and the formation of germinal centers in the thymus (15, 16). Myasthenia gravis patients often go through thymectomy, a procedure that shows an overall improved clinical outcome, highlighting the contribution of thymic abnormalities to the production of autoantibodies against the acetylcholine receptor and severity of the disease (17–19). Recent evidence suggests that infiltration of B cells to the thymus and thymic stroma destruction precedes type 1 diabetes development in NOD mice (20), supporting the idea that B cell infiltration to the thymus may be a common event in several autoimmune diseases. Alterations in the thymus structure have also been observed in rheumatoid arthritis and SLE, but the contribution of these abnormalities to these diseases are poorly understood (7, 21, 22). Among patients with SLE, between 1.5 and 2% develop thymomas and undergo thymectomy as treatment. In contrast to myasthenia gravis patients, this procedure on SLE progression has no clear health benefits (5, 9, 23).

Studies using BWF1 mice, a murine model of SLE, showed an increase in B cell frequency in the thymus of diseased mice compared to those that have not yet developed the disease (24, 25). In these studies, the authors highlight that the B1/B2 ratio in the thymus is higher than in spleen and blood. They showed that the B1 cell population migrates abnormally to the thymus due to high expression of the CXCR5 chemokine receptor, and aberrant high expression of CXCL13 (B lymphocyte chemoattractant, BLC) by myeloid dendritic cells present in the thymus. However, this study does not address the functional relevance of abnormal B cell numbers within the thymus and their contribution to SLE.

The converging evidence of B cells and plasma cells accumulation in the thymus during aging and particularly in autoimmune diseases prompted us to hypothesize that this lymphoid organ may become a specialized niche for B cells and plasma cells in the context of SLE development. To address this question, we characterized the B cell population of the thymus of BWF1 mice during the autoimmune humoral response. Here we show that upon the onset of the disease, the thymus structure becomes highly disorganized, exhibiting an increasing number of B cells that accumulate into structures that resemble ectopic germinal centers. Accordingly, we observed the presence of antibody-secreting plasma cells, a fraction of which produces anti-dsDNA autoantibodies. Noticeably, we further found that thymic B cells from diseased BWF1 mice induce the activation and differentiation of CD4+ thymocytes to follicular helper T cells. Altogether these data suggest a positive feedback loop, where thymic B cells induce the differentiation of follicular helper T cells that in turn promote the differentiation of autoreactive plasma cells in the thymus.

## Materials and Methods

### Mice

Female lupus-prone [NZB × NZW]F1 (BWF1) mice were purchased from Jackson Laboratory (Bar Harbor, ME, USA) and maintained at the animal facility of Fundacion Ciencia & Vida. Animal work was carried out under the institutional regulations of the Fundacion Ciencia & Vida and was approved locally by the ethical review committee of the Facultad de Ciencias, Universidad de Chile. Disease incidence and severity was monitored by measuring proteinuria using a standard semi-quantitative Combur Test N (Roche Diagnostics, Germany) and an ELISA to determine antibody titers to double-stranded DNA (dsDNA). To detect early autoimmune disease, proteinuria was measured monthly during the first 5 months of age and every week after that. In this work, we used 3 and 5 months old BWF1 female mice as young mice which still do not develop autoimmune disease. Diseased mice were 9 months old in average, presented severe proteinuria (i.e., ≥500 mg/dL protein) and high levels of plasmatic antibody titers against double-stranded DNA. In all cases, age-matched [NZW × BALB/c]F1 female mice were used as non-autoimmune controls.

### Flow Cytometry and t-SNE

Cell surface staining was performed in ice-cold PBS with 2% fetal bovine serum (FBS) for 30 min in the presence of Fcγ R blocking antibody (CD16/32). Viability dye eFluor 780 reagent (eBioscience) or propidium iodide (PI) were used for live/dead cells discrimination. Monoclonal antibodies (mAbs) against mouse CD8 (53-6.7) FITC, CD138 (281-2) PE or BV421, CD45R/B220 (RA3- 6B2) APC or PE-Cy7, CD19 (6D5) FITC, APC or eFluor 780, CD44 (IM7) APC, CD69 (H1.2F3) PE, CD83 (Michel-19) FITC, CD86 (GL1) FITC, IgM (RMM-1) PE-Cy7, purified CD16/32 (93), CXCR5 (L138D7) PE, Ki-67 (11F6) Alexa fluor 488, OX40L (RM134L) Alexa fluor 647, Blimp-1 (5E7) PE, Bcl-6 (IG191E/A8) Alexa fluor 647, and IgG-HRP (polyclonal) were purchased from BioLegend (San Diego, CA, USA). mAbs against mouse IgD (11-26c.2a) FITC, CD5 (53-7.3) PE-Cy7, CD21/35 (4E3) PE, GL7 (GL7) eFluor 660, CD11c (N418) PE, CD62L (MEL-14) FITC, CD25 (PC61.5) APC, CD8 (53-6.7) APC-eFluor 780, CD103 (2E7) FITC, CD279/PD-1 (J43) FITC, and Foxp3 (FJK-16s) PE-Cy7 were purchased from eBioscience (San Diego, CA, USA). mAbs against mouse I-Ad FITC (AMS- 32.1), were purchased from BD Pharmingen (San Diego, CA, USA). Intracellular staining for Foxp3 and Bcl-6 was performed after cell surface staining using the Foxp3/Transcription Factor Staining Buffer Set (eBioscience) following the manufacturer's instructions. Flow cytometry was conducted on a FACSCanto II flow cytometer (BD Biosciences) or FACSAria III (BD Biosciences) and data analysis was performed using the FlowJo software version 8 (Tree Star, Inc., Ashland, OR, USA).

For t-SNE, data was acquired in a FACS Aria III (BD Biosciences) and the analysis was performed using the Rtsne package in R software. Cells were pre-gated in FlowJo v10 (Tree Star) on single cells, live (PI negative), CD45+, CD3+, and CD4+/CD8–. After gating, 15,000 cells from both control and BWF1 mice were used as input for the tSNE analysis and the parameters were set to 1,000 iterations, theta 0.5, learning rate 200 and perplexity 30.

### ELISpot

Millipore^®^ MAIPS4510 96-well-plates were activated for 2 min with 50 μl/well of 70% ethanol and washed five times with deionized water. Plates were coated with 15 μg/ml of capture antibody anti-mouse IgG or dsDNA at 10 μg/ml and incubated overnight at 4°C. The plates were pre-treated with 10 μg/ml of methyl-BSA for 3 h at 37°C to evaluate reactivity against dsDNA. Subsequently, the plates were washed with PBS and blocked with RPMI medium supplemented with 10% FBS. The number of viable cells was carefully determined and plated in triplicates. After incubation at 37°C, 5% CO2 for 22 h, the plates were washed five times with PBS and added 0.5 μg/ml of biotinylated goat anti-mouse IgG and incubated for 2 h at room temperature. Then plates were washed five times with PBS, and avidin-enzyme conjugated to HRP (eBioscience) was added and incubated for 1 h at room temperature. After washing the plates five times with PBS, 3-amino-9-ethyl carbazole (AEC) substrate was added and incubated at 37°C for 30 min. The plates were washed with bidistilled water and dry uncovered for 3 h at 37°C. Plates were read using an ELISpot reader AELVIS and the software Eli.Analyse ELISPot Analysis Software V6.0.

### Confocal Microscopy

Thymi were extracted from diseased BWF1, and age-matched control animals, thymus lobes were imbibed in RPMI + 10% FBS + 5% low melting point agarose (Invitrogen) solution. Once the agar solidified at room temperature, slices of 400 μm were obtained in a PELCO^®^ 102 vibratome. The slices were fixed with 3.7% paraformaldehyde for 20 min at room temperature and stained for 30 min at 37°C with the following antibodies: CD4 (RM4-5) PE, CD8 (53 6.7) FITC, and CD19 (1D3) APC. After that, slices were washed with PBS, and placed on a slide with ProLong Gold antifade mounting medium (Invitrogen) and covered with a coverslip. Thymic slices were analyzed in the Zeiss LSM 710 confocal microscope, and the images analyzed with ImageJ.

### Immunohistochemistry

Thymi were extracted from diseased BWF1, and age-matched control animals, and frozen at −80°C for 24 h in OCT compound. Six micrometer cryostat sections were obtained, air dried and fixed in cold acetone for 15 min. Sections were then incubated with a single drop of peroxidase blocker for 7 min at room temperature, washed with PBS and incubated for 1 h with blocking solution (PBS + 1% BSA + 10% goat serum). Then, sections were incubated overnight at 4°C with anti-mouse B220 or anti-mouse cytokeratin 5. After that, the sections were incubated for 1 h with HRP-coupled secondary antibody, washed with PBS and incubated with DAB (3′-Diaminobenzidine) for 4–8 min. Finally, sections were stained with hematoxylin and dehydrated to be mounting and visualized in the Olympus BX51 microscope.

### Coculture of Follicular Helper T Cells With B Cells

Follicular helper T cells (T_FH_) and B cells of thymus and spleen were isolated by cell sorting from diseased BWF1 mice. T_FH_ were stained with antibodies to CD4, CD8, PD-1, CXCR5, and selected as CD4^+^CD8^−^PD-1^+^CXCR5^+^. B cells were purified as CD19^+^CD5^−/int^. CD4^+^ T cells that do not express markers of T_FH_ (PD-1^−^CXCR5^−^) were used as control. Dead cells were discarded using PI staining during the sorting. B cells were stained with CellTrace Violet (Invitrogen) according to the manufacturer's instructions and cocultured with T_FH_ at a 5:1 ratio (50,000 B cells and 10,000 T_FH_ per well). Cocultures experiments were maintained for 5 days at 37°C and 5% CO_2_. Subsequently, cells were recovered, and live B cells analyzed for GL-7 expression and dilution of CellTrace Violet stain by flow cytometry.

### Coculture of B Cells With Thymocytes to Assess T_FH_ Differentiation

Thymic B cells from diseased BWF1 and age-matched control mice were isolated by cell sorting (CD19^+^CD5^−/int^CD11c^−^) while thymocytes (of 3 m-control mice) were isolated as I-Ad negative cells (to deplete antigen presenting cells). Dead cells were discarded using PI staining during the sorting. After sorting, thymocytes were stained with CellTrace Violet (Invitrogen) according to the manufacturer's instructions and cocultured with B cells at a 10:1 ratio (100,000 thymocytes and 10,000 B cells) in presence of rmIL-7 (6 ng/ml-eBioscience). The cells were cultured in RPMI medium supplemented with 10% FBS, 0.055 μM 2-mercaptoethanol (Gibco), and 0.5 μg/ml Fungizone (Gibco), in U-bottom 96-well-plate (Falcon^®^). In some cases, a blocking antibody against OX40L (BioLegend, clone RM134L, 10 μg/mL) was used during the co-cultures. Coculture was maintained for 5 days at 37°C and 5% CO_2_. Subsequently, cells were recovered, and thymocytes analyzed for T_FH_ phenotype (CD4^+^CD8^−^PD-1^+^CXCR5^+^) in a PI negative gate by flow cytometry.

### *In vitro* B Cell Activation

Total thymic cells were activated at 2 × 10^6^ cells/ml with anti-mouse CD40 at 1.5 μg/ml and anti-mouse IgM at 5 μg/ml for 5 days at 37°C and 5% CO_2_ to evaluate OX40L expression on B cells by FACS. On the other hand, total thymic cells were activated at 2 × 10^6^ cells/ml with LPS at 2 μg/ml for 3 days at 37°C and 5% CO_2_ to evaluate Blimp-1 expression on B cells after fixation permeabilization with the corresponding buffer.

### RNA-Sequencing

RNA extraction from thymic B cells was carried out on 0.6 × 10^6^ thymic B cells isolated by cell sorting (CD19^+^CD5^−/int^CD11c^−^) recovered directly in 0.5 ml of TRIzol reagent (Life Technologies). Quantification of RNA was performed using specific fluorometry with the Qubit RNA quantification assay (Life Technologies). RNA integrity was assessed using an RNA Quality Measurement Number (RQN) of Fragment analyzer with the High Sensitivity RNA Analysis Kit (Advanced Analytical Technologies), were used RNA samples with RQN values >8.2. Sequencing libraries were prepared using the KAPA Stranded mRNA-Seq kit according to the manufacturer's protocol (Illumina). The length of the libraries was determined by capillary electrophoresis using the Standard Sensitivity NGS Fragment Analysis kit (Advanced Analytical Technologies). Libraries were quantified using the KAPA Library Quantification Kit (Kappa Biosystem) using the Eco PCR system (Illumina), following manufacturer's protocol. Libraries were sequenced on a Miseq platform (Illumina) using a v3 150 kit with 2 × 75 bp paired-end. Samples were subsequently analyzed using R/Bioconductor, and the DESeq2 procedure was used to normalize the data. Differentially expressed genes were identified using an adjusted *p*-value cut-off of 0.05 and a fold change of at least 1.5.

The data discussed in this publication have been deposited in NCBI's Gene Expression Omnibus and are accessible through GEO Series accession number GSE147359 (https://www.ncbi.nlm.nih.gov/geo/query/acc.cgi?acc=GSE147359).

### Apoptosis Assay

Cells isolated from the thymus of BWF1 diseased and age-matched control mice were stained with 1 μl of Annexin V FITC (BioLegend) and propidium Iodide at 1 μg/ml in 100 μl of binding buffer (HEPES 10 mM, NaCl 140 mM, CaCl_2_ 2.5 mM) by incubating for 15 min at room temperature. Staining was stopped by adding 200 μl of binding buffer, and the cells were analyzed by flow cytometry.

### Statistical Analysis

Statistical analysis was performed with the GraphPad Prism program V6 (GraphPad Software, San Diego, CA, USA). The data were compared using a Student's *t*-test after verification of normal distribution. Mann Whitney test was used when the data did not adjust to a normal distribution. Wilcoxon signed-rank test was used to compare data with hypothetical value. *P* < 0.05 were considered significant.

## Results

### Increased B Cell Numbers in the Thymus of Diseased-BWF1 Mice Correlates With an Abnormal Thymic Structure

**Figure 1:**
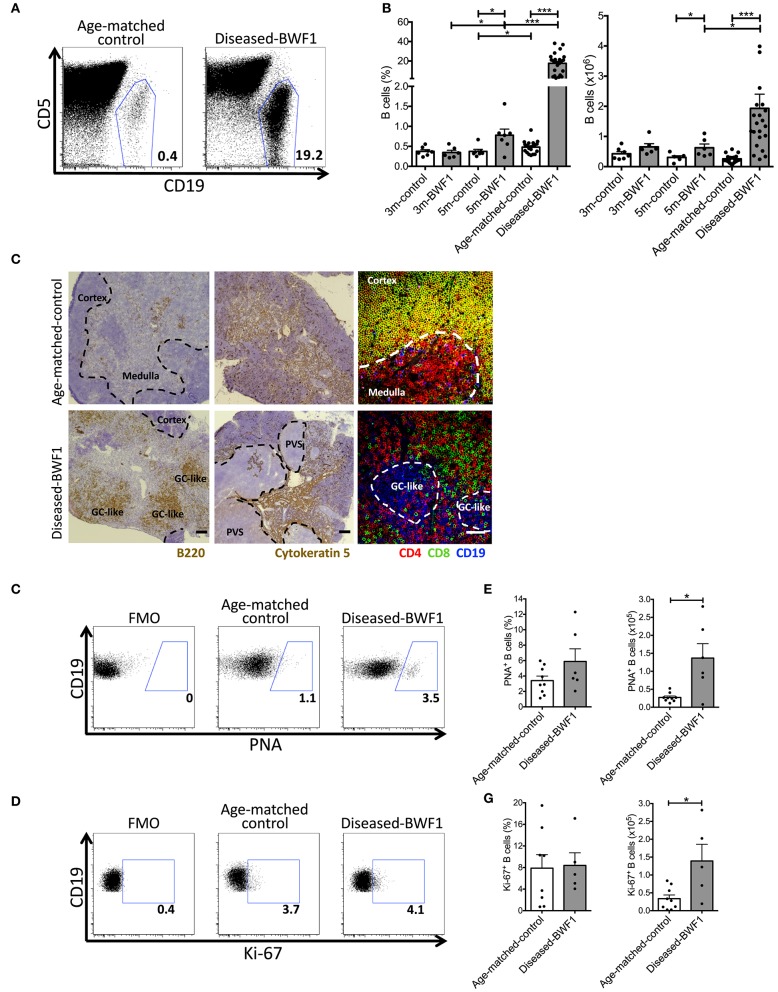
Diseased-BWF1 mice present proliferating thymic B cells that cluster in germinal center-like structures. **(A)** Representative FACS analysis of CD19^+^ cells in the thymus of diseased-BWF1 and age-matched-control mice. CD5 was used to exclude T cells. **(B)** Thymic B cell frequency (left) and absolute number (right) in BWF1 mice at different ages prior (3 and 5 months old) and after the onset of the disease and age-matched control mice. B cells were analyzed as CD19^+^CD11c^−^CD5^−/int^ cells. Each dot represents one mouse (*n* = 6–20 mice per group). Student's *t*-test, ^*^p ≤ 0.05; ^***^p ≤ 0.001. **(C)** Representative light microscopy images of B220 (left panel) and cytokeratin 5 (middle panel) staining of thymic tissue from diseased-BWF1 and age-matched-control mice. Scale bar: 200 μm. PVS: perivascular spaces. GC-like: germinal center-like structures. The right panel shows representative confocal microscopy images of CD4^+^ T cells (red), CD8^+^ T cells (green), and CD19^+^ B cells (blue) in thymic tissue of BWF1-disease and age-matched control mice. Scale bar: 50 μm. **(D)** Flow cytometry plots of PNA expression in thymic B cells (CD19^+^CD11c^−^CD5^−/int^ gate) from diseased-BWF1 and age-matched-control mice. **(E)** Frequency and absolute number of PNA^+^ B cells in the thymus of diseased-BWF1 and age-matched-control mice. Mann-Whitney test, *p* ≤ 0.05. **(F)** Flow cytometry plots of Ki-67 expression in thymic B cells from diseased-BWF1 and age-matched-control mice **(G)** Frequency and absolute number of Ki-67^+^ B cells (CD19^+^CD11c^−^CD5^−/int^ gate) in the thymus of diseased-BWF1 and age-matched-control mice. Student's *t*-test, *p* ≤ 0.05. Data represent 3–4 independent experiments.

We and others have reported that the frequency of B cells in the thymus increases significantly during normal aging and in several autoimmune diseases (4, 14, 20, 26). We aimed at studying the dynamics of B cell accumulation in the thymus of BWF1 lupus-prone mice during the development of the autoimmune response. For this, we analyzed the thymus of BWF1 mice at different stages preceding (3 and 5 months old) and after the onset of the disease (9 months old in average) and compared the results to age-matched controls (NZWxBALB/c)F1 mice. We observed a higher than 20-fold increase in B cell frequency and a significant 6-fold increase in absolute B cell numbers in the thymus of diseased-BWF1 mice compared with age-matched control mice (Figures 1A,B). A modest but significant increase in the frequency of B cells in the thymus of 5 months old BWF1 mice compared to 3 months old BWF1 mice suggest that the frequency of B cells present in the thymus increases before the onset of proteinuria.

Histological examination of the thymus of diseased-BWF1 and age-matched control mice revealed remarkable alterations in the structure of the thymus at the onset of the disease (Figure 1C). Fluorescent co-staining of CD4, CD8, and CD19 confirmed that the thymus of diseased mice is characterized by the presence of large B cell clusters and the absence of CD4^+^CD8^+^ double- positive (DP) thymocytes (Figure 1C) which are normally found in the cortex (27). In diseased mice (9 months old on average), there was a reduction of the cortex areas and large numbers of B cells (B220^+^) clustered into structures reminiscent of germinal centers. In contrast, in age-matched control mice, we observed small numbers of B cells disseminated within the medulla (Figure 1C), as previously reported (11). Next, we analyzed the expression of the germinal center marker PNA on thymic B cells (Figure 1D). Although we found no statistical difference in the frequency of PNA^+^ B cells between age-matched control and diseased BWF1 mice, the absolute number of PNA^+^ B cells increase 5-fold in diseased BWF1 mice compared to control mice (Figure 1E). We also found an increase in the absolute number, but not in the frequency, of Ki67^+^ B cells in the thymus of diseased BWF1 mice (Figures 1F,G). Since we do not observe an increase in the percentage of Ki67+ B cells, our data suggest that the expansion of B cells in the thymus of autoimmune mice may be due to an increase in the migration of B cells from the periphery. Interestingly, we found that in diseased mice there was an expansion of non-epithelial perivascular spaces (PVS) (cytokeratin-5^−^) where most B220^+^ B cells clustered (Figure 1C, Supplememtary Figure 1). Altogether these results demonstrate that at the onset of SLE, the thymus of BWF1 mice undergoes remarkable changes in terms of structure and B lymphocyte content, with the appearance of ectopic germinal center-like structures.

### The Thymus of Diseased-BWF1 Mice Harbors IgG Anti-dsDNA Antibody-Secreting Plasma Cells

Present evidence indicates that germinal center formation depends on the activation of antigen-specific B cells by cognate T cells leading to the formation of antibody-secreting plasma cells and memory B cells (28, 29). The distribution of B cells in germinal center-like structures in the thymus of diseased-BWF1 mice suggests that they may be locally activated and differentiated into memory B cells or plasma cells. We next characterized the thymic B cells by analyzing their expression of differentiation markers. Analysis of isotype switched (IgM^−^IgD^−^) memory B cells in diseased mice did not show a significant difference compared to control mice, whereas diseased mice present a significant increase in naïve B cells (IgM^+^IgD^+^) compared to control mice (Supplementary Figure 2). These data indicate that a substantial fraction of B cells accumulating in the thymus of diseased mice rather display a naive than a memory phenotype.

**Figure 2:**
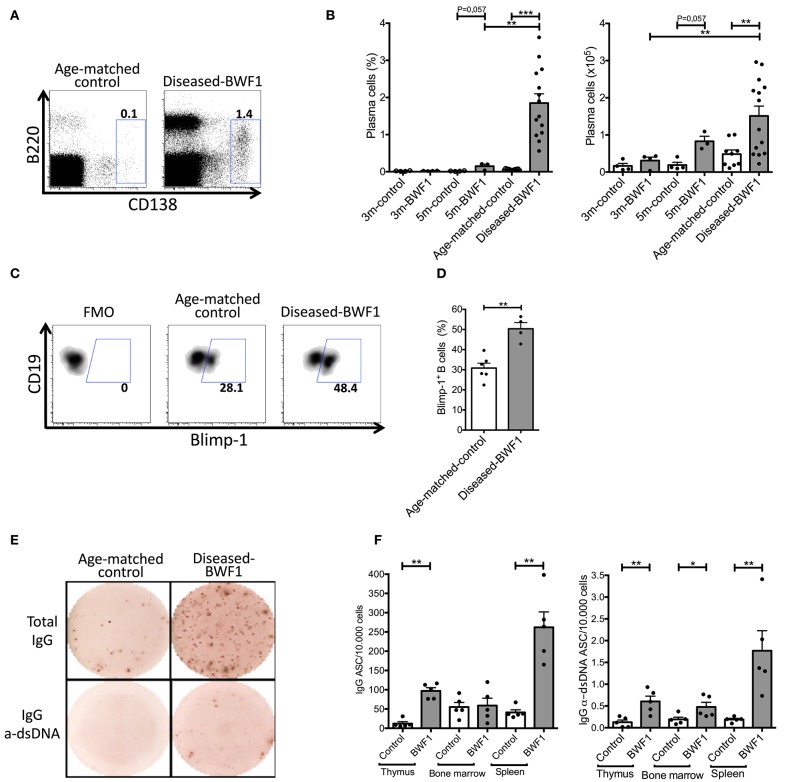
The thymus of diseased-BWF1 mice harbors IgG anti-dsDNA antibody-secreting plasma cells. **(A)** Flow cytometry plots of B220^int^CD138^+^ plasma cells gated on live thymic cells of diseased-BWF1 and age-matched-control mice. **(B)** Frequency (left) and absolute number (right) of plasma cells (B220^int^CD138^+^) in BWF1 mice and age-matched controls at different stages prior and after the onset of the disease. Each dot represents one mouse (*n* = 3–14 mice). **(C)** Flow cytometry plots and **(D)** frequency of Blimp-1^+^ thymic B cells from diseased-BWF1 and age-matched control mice after activation with LPS. Each dot represents one mouse (*n* = 4–6 mice). Mann-Whitney's, ^**^p ≤ 0.01. **(E)** Representative ELISPOT of total and anti-dsDNA IgG antibody-secreting cells (ASC) present in the thymus of diseased-BWF1 and age-matched control mice. **(F)** Quantification of total and anti-dsDNA IgG antibody-secreting cells in the thymus, bone marrow and spleen from diseased-BWF1 and age-matched control mice. Each dot represents one mouse (*n* = 5 mice). Student's *t*-test, ^*^p ≤ 0.05; ^**^p ≤ 0.01; ^***^p ≤ 0.001.

Interestingly, the analysis of thymic plasma cells revealed a significant increase in the percentage and absolute number of plasma cells (B220^int^CD138^+^) in the thymus of diseased-BWF1 mice compared to age-matched control animals (Figures 2A,B). These results are consistent with a higher percentage of Blimp-1^+^ B cells, a transcription factor driving the differentiation of B cells to plasma cells (30, 31), in diseased mice compared to control mice (Figures 2C,D). To investigate the presence of functional plasma cells and their specificity, we enumerated antibody-secreting cells (ASC) by ELISpot. These experiments revealed that the thymi from diseased-BWF1 mice contain significantly higher numbers of IgG ASC (>5 times) compared to those from age-matched control mice (Figures 2E,F). When compared to IgG production from other organs known to harbor ASC such as bone marrow and spleen, we found a comparable number of spots of IgG ASC between the thymus and bone marrow in diseased-BWF1 mice (Figure 2F).

Of note, the thymus of diseased-BWF1 mice contained few IgM ASC, and we could not detect anti-dsDNA ASC of the IgM class (Supplementary Figure 3). Thus, the majority of thymic plasma cells in diseased BWF1 mice have gone through isotype switching secreting mainly IgG antibodies, some of which are specific of dsDNA. In summary, our results show that autoimmune BWF1 mice have proliferating B cells in germinal center-like structures within the thymus, which most likely support the differentiation of B cells into anti-dsDNA IgG-secreting plasma cells. Thymic ASC might, therefore, contribute in a significant way to the pool of secreted IgG autoantibodies found in autoimmune mice.

### Thymic B Cells From Diseased-BWF1 Mice Express Genes Associated to Cell Survival

**Figure 3:**
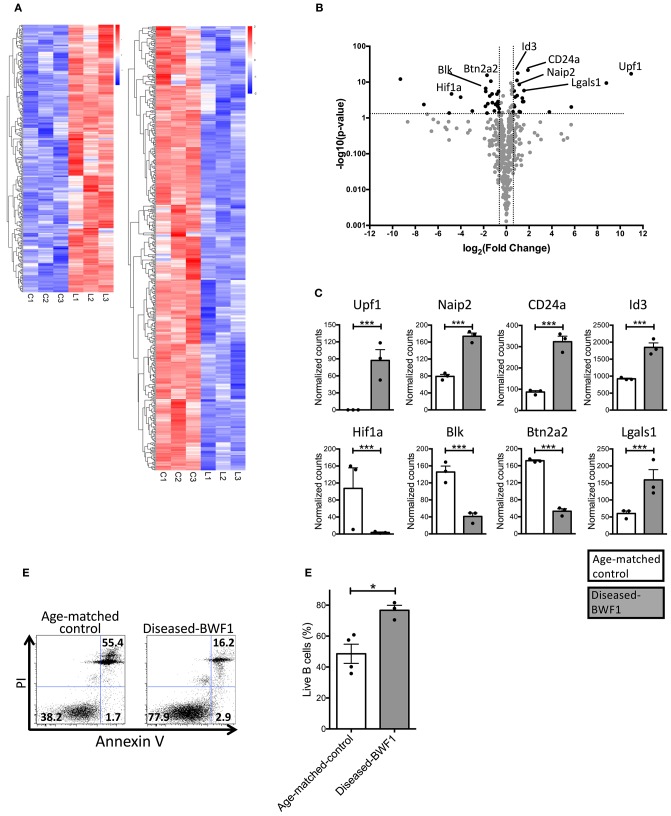
Thymic B cells from diseased-BWF1 mice present a transcriptional profile associated to cell survival. **(A)** RNAseq transcriptome analysis of thymic B cells from diseased-BWF1 and age-matched control mice. Heatmap shows genes that are upregulated (red) and downregulated (blue) with at least 1.5-fold change and adjusted *p* < 0.05 (detailed list of genes is provided in Supplementary Tables 1, 2). **(B)** Volcano plot of selected genes related to apoptosis and B cell development. **(C)** RNA-seq normalized counts for selected genes. White bars: age-matched control mice; gray bars: diseased BWF1 mice. **(D)** Flow cytometric analysis of live and apoptotic B cells as assessed by Annexin V and PI in cells from thymus of diseased-BWF1 mice and age-matched control mice. **(E)** Frequency of live thymic B cells (Annexin V^−^PI^−^) from diseased-BWF1 mice and age-matched control mice. Mann-Whitney's *t*-test, ^*^p ≤ 0.05, ^***^p ≤ 0.001.

Our data show that the B cells found in the thymus of diseased mice are distinct from normal resident thymic B cells in terms of abundance, localization, proliferation, and antibody secretion. To gain insights into the mechanisms that lead to the accumulation of this peculiar B cell population as well as into their function(s), we analyzed their transcriptomic profile using RNAseq. We identified 337 upregulated genes and 492 downregulated genes in thymic B cells from diseased-BWF1 mice compared to age-matched control mice (Figure 3A, Supplementary Tables 1, 2). Among the upregulated genes, several were related to B cell survival and development including Upf1 and Naip2 (Figure 3B). Also, CD24a, a negative regulator of early pre-B cell differentiation in the bone marrow was likewise upregulated in diseased mice (32–35). Among the genes that were downregulated in thymic B cells from diseased-BWF1 mice, we found Hif1a, Blk, and Btn2a2, whose low expression has been associated with the induction or development of several autoimmune diseases such as collagen-induced arthritis, experimental autoimmune encephalomyelitis and SLE (36–39). The normalized counts of these genes are shown in Figure 3C. To confirm that thymic B cells obtained from diseased BWF1 mice have enhanced survival compared to B cells from age-matched controls, we assessed live B cells through Annexin V/PI assay. As shown in Figures 3D,E, the live fraction of thymic B cells was significantly higher in diseased-BWF1 mice, suggesting that the thymus of diseased-BWF1 mice provides a niche that supports the survival of B cells.

### The Thymus of Diseased-BWF1 Mice Harbor Functional Follicular Helper T Cells

**Figure 4:**
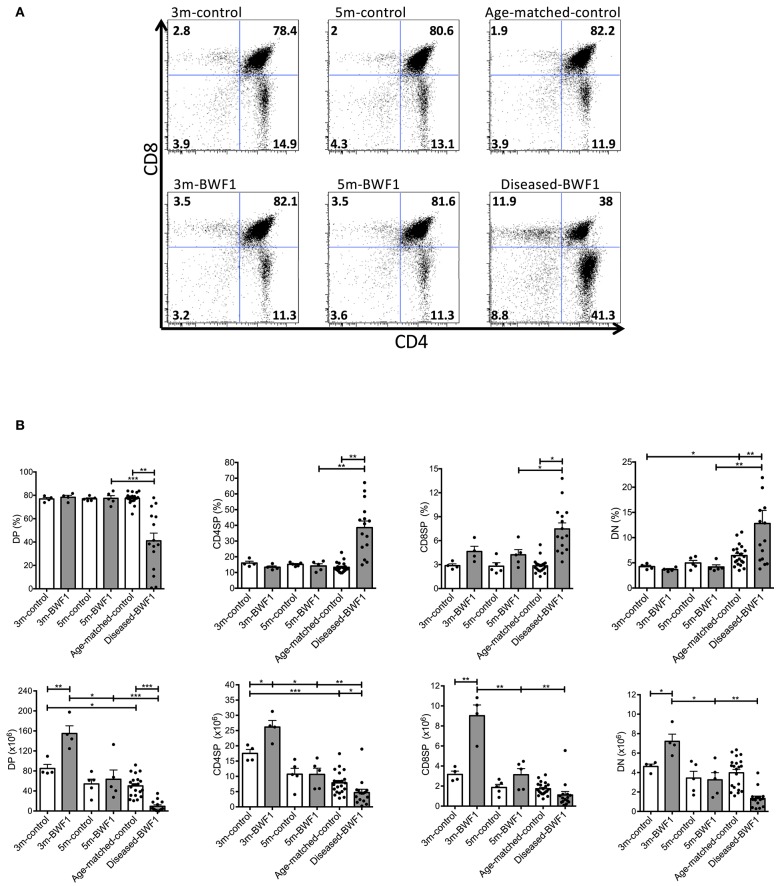
Subpopulations of thymocytes are altered in diseased-BWF1 mice. **(A)** Representative FACS analysis of thymic DP, CD4SP, CD8SP, and DN populations in BWF1 mice at different ages prior and after the onset of the disease and age-matched control mice. The cells were analyzed in a I-Ad^−^ gate (*n* = 4–15 mice per group). **(B)** Summary of the frequency (up) and the absolute number of thymocyte populations (down). Each dot represents one mouse. Student's *t*-test, ^*^p ≤ 0.05; ^**^p ≤ 0.01; ^***^p ≤ 0.001.

In addition to the abnormal thymic structure and accumulation of B cells, characterization of the T cell thymic compartment of diseased BWF1 mice showed a significant reduction of CD4^+^CD8^+^ double-positive (DP) thymocytes compared to 3 and 5 month-old BWF1 mice (Figure 4), which is consistent with these animals exhibiting a smaller cortex, as observed by histology (Figure 1C). Along with the decrease of DP cells, we found an increase in the frequency of CD4^−^CD8^−^ double-negative cells (DN) and both CD4^+^ and CD8^+^ single-positive (SP) thymocytes compared to 3 and 5 month-old BWF1 mice and control mice (Figure 4). These results indicate that during the onset of the disease, the thymus of BWF1 autoimmune mice suffers significant changes in its T cell composition in addition to the accumulation of B cells. CD69 expression on DP cells was only transiently decreased in 3 months-old BWF1 mice, previous to developing proteinuria, suggesting that the process of positive selection is not altered during the disease (Supplementary Figure 4).

**Figure 5:**
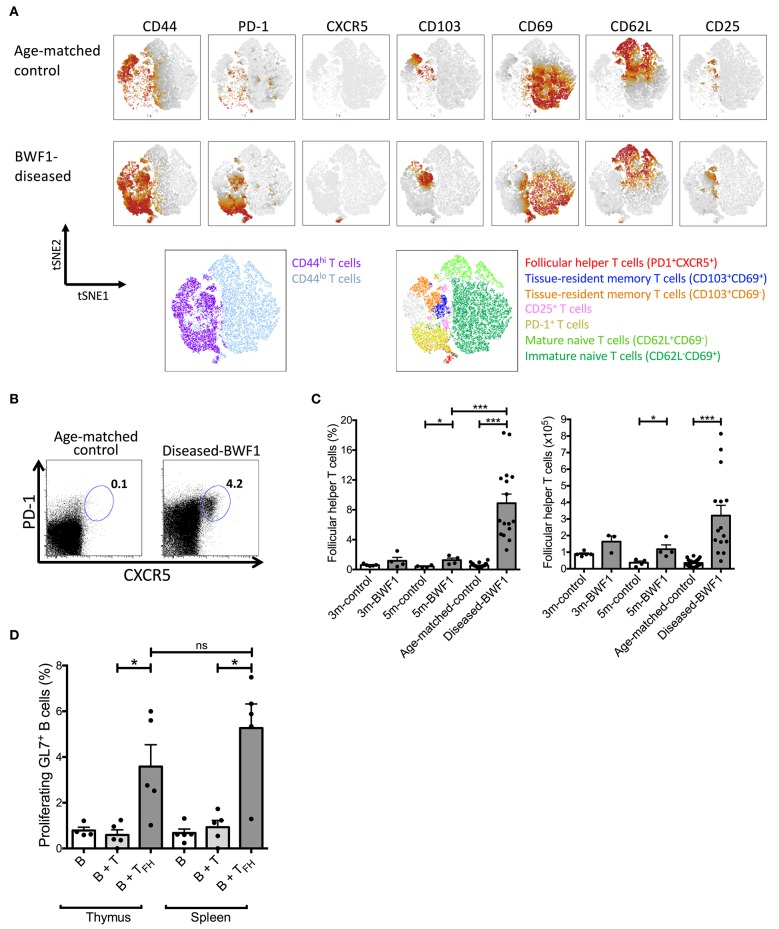
Functional follicular helper T cells increase in the thymus of diseased-BWF1 mice. **(A)** tSNE analysis of CD4SP (CD4^+^CD8^−^ gate) cells of diseased-BWF1 and age-matched-control mice. Bottom panel shows populations colored by manual gating. **(B)** Flow cytometry plots of PD-1 and CXCR5 expression in CD4SP cells from the thymus of diseased-BWF1 mice and age-matched-control. **(C)** Frequency and absolute number of follicular T helper cells (PD-1^+^CXCR5^+^) in BWF1 mice at different ages prior and after the onset of the disease and age-matched control mice. Each dot represents one mouse (*n* = 4–16 mice). Student's *t*-test, ^*^p ≤ 0.05; ^***^p ≤ 0.001. **(D)** Percentage of proliferating thymic GL7^+^ B cells (CD19^+^) after coculture with either PD-1^+^CXCR5^+^ thymic follicular helper T cells (B+T_FH_) or thymic PD-1^−^CXCR5^−^ non-follicular CD4^+^ T cells (B+T) isolated from diseased-BWF1 mice. The same experiments were performed with cells from spleen. Each dot represents one mouse (*n* = 4–5 mice). Student's *t*-test, ^*^p ≤ 0.05.

Interestingly, transcriptomic profiling of thymic B cells from diseased BWF1 mice further revealed the increased expression of Id3 and Lgals1 (Figures 3B,C), two genes known to support the maintenance of germinal center B cells and humoral immune response (40–42). Therefore, we investigated whether the frequency and number of follicular helper T (T_FH_) cells were enhanced in the thymus of diseased-BWF1 mice. tSNE analysis on CD4^+^ T cells allowed us to dissect the composition of the naive (CD44^lo^) and antigen experienced (CD44^hi^) T cell thymic compartments. As shown in Figure 5A, Supplementary Figures 5A,B, we observed an abundance of antigen-experienced CD44hi T cells in the thymus from diseased-BWF1 mice compared to age-matched control mice. The increase in antigen experienced T cells in diseased mice is concomitant to a reduction in the immature and mature naïve T cells (Supplementary Figures 6A,B). Among thymic antigen-experienced CD4+ T cells found in diseased-BWF1, we detected a variety of different subsets such as memory tissue-resident CD103^+^, some of which also express CD69. We also observed an increase in the frequency of regulatory T cells in diseased mice (Supplementary Figures 6A,B). Moreover, tSNE analysis revealed the appearance in diseased mice of a subset with T_FH_ phenotype co-expressing PD-1 and CXCR5, which was absent in age-matched controls (Figure 5A). Further analysis revealed that the percentage and absolute numbers of PD-1^+^CXCR5^+^ increased dramatically in the thymus of diseased BWF1 mice compared to the thymus of age-matched control mice (Figures 5B,C). These thymus-residing PD-1^+^CXCR5^+^ cells express high levels of Bcl-6, a transcription factor that specifies T_FH_ program (Supplementary Figure 7). These results suggest that the presence of B cells and GC-like structures in the thymus of diseased BWF1 mice may be associated to the appearance of T_FH_ cells.

Similar to the role of T_FH_ cells in B-cell maturation during normal immune responses, results from animal models of SLE as well as from patients with this disease indicates that T_FH_ cells are required for autoantibody production (43, 44). To evaluate if T_FH_ present in the thymus of diseased mice could drive B cell activation and proliferation, we performed *in vitro* co-culture assays with sorted thymic T_FH_ and B cells from diseased BWF1 mice (45). Thymic T_FH_ from diseased BWF1 mice induced the proliferation of activated GL7+ B cells to a similar level as splenic T_FH_ obtained from BWF1 mice (Figure 5D). Of note, non-T_FH_ CD4^+^ T cells from either thymus or spleen were unable to induce proliferation of activated B cells (Figure 5D). These results indicate that T_FH_ present in the thymus of autoimmune mice are functional and possibly contribute to the activation and expansion of thymic B cells in diseased BWF1 mice.

### Thymic B Cells From Diseased BWF1 Mice Induce the Differentiation of Follicular Helper T Cells

**Figure 6:**
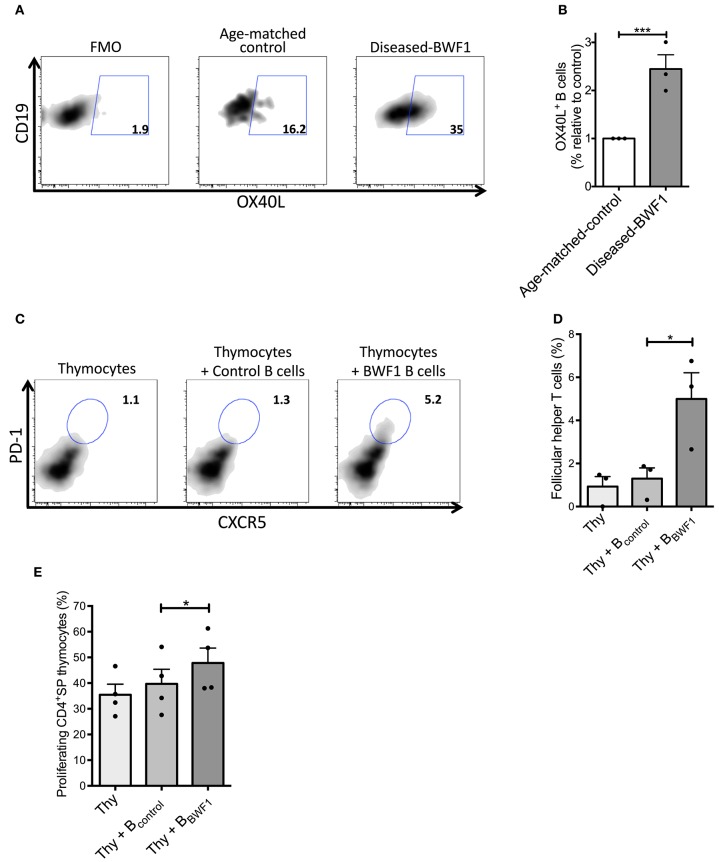
Thymic B cells favor the expansion of follicular helper T cells in diseased-BWF1 mice. **(A)** Flow cytometry plots of OX40L expression in thymic B cells from diseased-BWF1 and age-matched-control mice following 5-days activation with anti-IgM and anti-CD40. **(B)** Frequency of thymic OX40L^+^ B cells from diseased-BWF1 relative to OX40L^+^ B cells from age-matched-control mice. Data represent three independent experiments. Wilcoxon signed rank's test, ^***^p ≤ 0.001. **(C)** Flow cytometry plots and **(D)** frequency of PD-1^+^CXCR5^+^ follicular helper T cells (in a CD4^+^CD8^−^ gate) 5 days after co-culture of thymocytes (Thy) with thymic B cells from diseased-BWF1 (Thy+B_BWF1_) or age-matched control mice (Thy+B_control_), in presence of IL-7 (6 ng/ml). Data shows representative results of three independent experiments. **(E)** Proliferation of CD4SP thymocytes assessed by cell-trace violet dilution after co-culture of control thymocytes (Thy) with thymic B cells from diseased-BWF1 (Thy+B_BWF1_) or age-matched control mice (Thy+B_control_). Data represent the results of three independent experiments. ^*^p ≤ 0.05.

It is known that B cells support T_FH_ cell differentiation via OX40L in the spleen (46, 47). This evidence prompted us to investigate whether thymic B cells could promote the differentiation of thymic T_FH_ cells. Accordingly, we found an increase in the frequency of thymic B cells expressing OX40L (2-fold) in diseased mice compared to age-matched controls (Figures 6A,B). Additionally, we did not find any differences in the expression of co-stimulatory molecules (CD83, CD86, and CD40) and the antigen presenting molecule (I-Ad) between B cells from diseased BWF1 mice and age-matched-control mice (Supplementary Figure 8). To demonstrate that thymic B cells favor the development of T_FH_ cells, we carried out co-culturing experiments of thymus B cells and thymocytes from control animals in the presence of IL-7. We observed that thymic B cells from diseased mice generate a more significant percentage of T_FH_ cells (PD-1^+^CXCR5^+^) than thymic B cells from age-matched control mice (5.2% with BWF1 B cells vs. 1.3% with control B cells) (Figures 6C,D). Splenic B cells were also are capable of inducing the differentiation of thymocytes to T_FH_ (Supplementary Figure 9). Of note, thymic B cells from diseased-BWF1 mice induced a higher proliferation of CD4^+^SP thymocytes than B cells from age-matched control mice (Figure 6E) but there were no differences in CD25 expression between the different conditions (data not shown). To evaluate whether OX40L is important to support the T_FH_ differentiation we performed the culture of thymic B cells from diseased-BWF1 mice with thymocytes in presence of an OX40L blocking antibody and we found a reduction on T_FH_ differentiation and proliferation of the CD4^+^SP thymocytes (Supplementary Figure 10). These results suggest that thymic B cells from diseased mice support the differentiation of thymocytes into T_FH_ cells through OX40L.

## Discussion and Conclusion

SLE is a chronic autoimmune disease of unknown etiology characterized by the formation of immune complexes, which are deposited in tissues causing inflammation. In SLE, both T and B cells are overactivated and recognize autoantigens related to nuclear proteins. Although the presence of B cells in the thymus in BWF1 mice, a murine model of SLE, has already been demonstrated (24, 25), the development of this population during the progression of this autoimmune disease remains mostly unexplored. Using the BWF1 mice, here we report an increase in the number and frequency of B cells, plasma cells, and follicular helper T cells (T_FH_) in the thymus of lupus diseased mice. Moreover, our data provide evidence that these B cells proliferate and cluster in ectopic germinal centers within the perivascular space (PVS) of the thymus. Additionally, thymic cells obtained from diseased mice produce IgG anti-dsDNA antibodies demonstrating the presence of autoantibody-secreting cells. Finally, we demonstrate that thymic B cells from diseased-BWF1 mice favor the differentiation of T_FH_, which may, in turn, promote the activation and differentiation of B cells into autoreactive plasma cells in the thymus.

Previous studies have shown the presence of B cells in the thymus, which has been attributed a role of antigen-presenting cells involved in the negative selection of T cells (10–12). However, we demonstrated that the thymus of diseased mice loses the classical structure defined by the functional separation in cortex and medulla, where the processes of selection of the T cells occur. This observation led us to investigate whether these B cells might be involved in different processes independent of antigen presentation and negative selection of T cells. In this line, Pinto and collaborators have reported that thymic B cells produce autoantibodies that attack the thymic stroma, an event that precedes the development of type 1 diabetes (20). Moreover, our previous results demonstrated that the human thymus, as it ages may provide a niche for viral-specific plasma cells (14). The novel data presented here showing the presence of ectopic germinal centers, auto-antibody secreting plasma cells, and T_FH_ cells strongly argue in favor of the idea that during an autoimmune response, the thymus may acquire a new function as a niche suitable for the development of a humoral immune response.

An important finding presented here is that during the development of the autoimmune response, there is a significant increase in the frequency of B cells present in the thymus of BWF1 mice. This is not only a consequence of enrichment of B cells due to the reduction of double-positive thymocytes since as we report, there is a 6-fold increase in the absolute number of thymic B cells in diseased-BWF1 mice. An unresolved question that arises from this work is the origin of the B cells that accumulate in the thymus of diseased-BWF1 mice. Adoptive transfer experiments with splenic B cells as well as experiments with parabionts have shown that migration of peripheral B cells to the thymus in steady-state conditions does not contribute significantly to the pool of thymic B cells (11, 48, 49). However, under inflammatory conditions such as systemic LPS treatment, *Candida albicans* or *Trypanosoma cruzi* infection, it was demonstrated that mature B and T cells could efficiently migrate to the thymus (50). Thus, an intriguing possibility is that during chronic inflammation in autoimmune BWF1 mice, B cells from the periphery may continuously migrate to the thymus where they survive, proliferate, and differentiate into plasma cells.

The central role of the CXCL13 chemokine (B lymphocyte chemoattractant or BLC) in the recruitment of B cells to the thymus has already been established (51). Using a murine model of myasthenia gravis, Weiss et al. demonstrated that although thymic overexpression of CXCL13 under steady-state condition does not induce B cell recruitment to the thymus, under inflammatory conditions such as after immunization with Poly (I:C), CXCL13 overexpression enhanced B cell migration to this organ (52). In the murine model of SLE, the group of Matsushima demonstrated that dendritic cells in the thymus of BWF1 mice produce CXCL13 which attracts B cells to this organ during the development of the disease (24). The same group further explored this possibility and showed that when B cells are injected intravenously, they can enter the thymic PVS and the medulla of aged BWF1 mice (25). Thus, CXCL13 production in the thymus under inflammatory conditions may be sufficient to drive B cell migration to this organ.

Further evidence of B cell lymphopoiesis within the thymus was previously reported by Perera et al. where the authors use the Rag2-GFP reporter mice and demonstrate that B cells can develop from precursors within the thymus (11). Thus, it may be possible that during the autoimmune response, B cell lymphopoiesis within the thymus might be enhanced or there might be an increase in the survival of B cells in this organ. Whether B cells come from the periphery or are differentiated *in situ*, our RNAseq data supports the idea that within the autoimmune thymus, B cells might be exposed to an altered environment that effectively boosts their proliferation and survival. Thus, in any possible scenario, the accumulation of B cells may be favored by remarkable changes in the thymic niche during the autoimmune response supporting B cell survival and/or differentiation.

Several studies support a role of IL-7 and Delta like 4 (Dll4)-Notch signaling pathways in regulating lymphocyte development in the thymus. The group of El-Kassar has demonstrated that transgenic mice that overexpress IL-7 show a dysregulation in thymocyte populations and an increase of B cell populations due to an induction of B -lymphopoiesis in the thymus. Interestingly, the treatment with IL-7 blocking antibodies reduces B cell populations in this organ (53). On the other hand, Billiard and collaborators have shown that anti-Dll4 treatment reduces thymocyte populations favoring the expansion of mature B cells in the thymus (54). Accordingly, it would be interesting to study IL-7 levels and Dll4 expression in the thymus of diseased-BWF1 mice in order to evaluate the contribution of these pathways in the aberrant cellular composition we observe in these mice.

The germinal center is a structure typically developed in secondary lymphoid organs where antigen-specific B cells receive the proper differentiation signals from T_FH_, proliferate, and undergo somatic hypermutation. Germinal centers form in the center of the B cell follicles of secondary lymphoid organs, interspersed within a network of stromal cells known as follicular dendritic cells (FDCs) (55, 56). The presence of ectopic germinal centers has been widely reported in the thymus of patients with myasthenia gravis, an autoimmune disease characterized by the presence of anti-acetylcholine receptor autoantibodies. In this disease, the development of thymic follicular hyperplasia is frequently observed, with the presence of ectopic germinal centers, characterized by the presence of T_FH_ cells, B cells, and FDCs (57, 58). Our own unpublished results show that the thymus of BWF1 diseased mice present CD21/CD35+ cells within the CD45- compartment, however, we cannot rule out that these cells are B cells with lower CD45 staining or a subset of thymic epithelial cells that express CD21/CD35 as self-antigens. Although here we present significant evidence that the thymus of BWF1 diseased mice also develops ectopic germinal centers (B cell organization in the tissue, presence of T_FH_ cells and plasma cells), further studies should elucidate if these structures resemble canonical lymph node germinal centers and demonstrate that thymic B cells undergo somatic hypermutation locally.

Using a different murine model of SLE, the B6*.Sle16* lupus-prone mice, it was reported that B cells support the generation of T_FH_ through OX40L expression (46). In that report, the authors demonstrate that the ablation of OX40L expression, specifically in B cells, results in the reduction of T_FH_ and a significant decrease in the autoimmune response in these mice. In agreement with a role of OX40L in the induction of T_FH_, we also show that in the diseased-BWF1 mice, there is an increase in the frequency of T_FH_ along with an increase in thymic B cells that express OX40L compared to control mice. Moreover, we demonstrate that only thymic B cells from diseased-BWF1 mice have the capacity to induce the differentiation of thymocytes to T_FH_ in an OX40L dependent fashion. Thus, our results recapitulate the role of OX40/OX40L interactions in the generation of the germinal center reaction observed in secondary lymphoid organs in lupus-prone mice reported by Cortini and collaborators (46). This leads us to propose that the germinal center reaction found in the thymus of lupus-prone mice is directly responsible for the generation of the auto-antibody secreting plasma cells in this organ rather that being the result of plasma cells arriving from the periphery. Activation of autoreactive B cells and differentiation into autoantibody producing plasma cells in germinal centers within the thymus may be favored by the interaction with T_FH_. Finally, thymic B cells may also induce the differentiation of CD4^+^ T cells to T_FH_, generating a positive feedback loop that sustains the humoral immune response within the thymus.

Interestingly, thymic morphological and functional alterations observed in the BWF1 and SLE patients have been described in several other autoimmune diseases including *myasthenia gravis*, type 1 diabetes, Sjogren's syndrome and ulcerative colitis (5, 20). Despite the distinct pathophysiological features, all these alterations have in common chronic inflammation, which may be hijacking the normal thymic function of T cell repertoire selection to establish a niche that sustains the humoral immune response. Studies on thymectomized BWF1 mice could give some insight into the role of the thymus as a source of autoantibodies and its real contribution to the development of the disease.

## Data Availability Statement

The datasets generated for this study can be found in the in NCBI's Gene Expression Omnibus and are accessible through GEO Series accession number GSE147359.

## Ethics Statement

The animal study was reviewed and approved by Comité de Bioetica de Fundacion Ciencia y Vida and CICUA from Universidad de Chile.

## Author Contributions

YH designed the study, performed experiments, analyzed the data, and wrote the manuscript. SN, MF, FF-S, PS, VM, and JD performed experiments and analyzed the data. MB, DS, SN, MR, EZ, and AL-D designed the study, analyzed the data, and wrote the manuscript. All authors critically read the manuscript.

## Conflict of Interest

The authors declare that the research was conducted in the absence of any commercial or financial relationships that could be construed as a potential conflict of interest.

## Supplementary Material

Supplementary Figure 1 Thymic B cells from diseased-BWF1 mice are localized in perivascular spaces. Representative images of thymic tissue from diseased-BWF1 stained with B220 (left panel) and cytokeratin 5 (right panel).

Supplementary Figure 2 Subpopulations of B cells are altered in diseased-BWF1 mice. **(A)** IgD and IgM expression in thymic B cells (CD19^+^GL7^−^) from diseased-BWF1 and age-matched-control mice. **(B)** Summary of the frequency (up) and absolute number (down) of the IgM^−^IgD, IgM^+^IgD^+^, and IgM^+^IgD^−^ B cells populations. ^**^*p* ≤ 0.01, ^***^*p* ≤ 0.001.

Supplementary Figure 3 Total IgM and IgM anti-dsDNA antibodies in thymus of diseased-BWF1 mice. ELISPOT analysis was performed with total cells obtained from the thymus of diseased-BWF1 and age-matched control mice. Representative experiment of two independent experiments.

Supplementary Figure 4 CD69 expression in DP thymocytes from diseased BWF1 and control mice. **(A)** Representative histogram of CD69 expression in DP thymocytes from diseased-BWF1 mice and age-matched- control mice. The analysis was performed in a CD4^+^CD8^+^(DP) gate. **(B)** Summary of CD69 expression in DP thymocytes of BWF1 mice at different ages prior and after the onset of the disease and age-matched control mice. MFI: Median fluorescence intense. **(C)** Summary of the frequency of CD69^+^ DP thymocytes from BWF1 mice at different ages prior and after the onset of the disease and age-matched control mice. Each dot represents one mouse (*n* = 3–6 mice per group). Student's *t*-test, ^***^*p* ≤ 0.001.

Supplementary Figure 5 Diseased-BWF1 mice present an increase in the frequency of antigen-experienced CD44^hi^ T cells in the thymus. Analysis of the thymic antigen-experienced T cells, immature, and mature naïve T cell population in BWF1 mice at different ages prior and after the onset of the disease and age-matched control mice **(A)** Representative example of CD44 and CD62L expression in thymocytes from diseased-BWF1 and age-matched control mice. Analysis was carried out in a CD4SP (CD4^+^CD8^−^) gate. **(B)** Frequency of thymic antigen-experienced T cell populations (CD44^hi^), immature (CD44^lo^CD62L^−^), and mature (CD44^lo^CD62L^+^) T cells in BWF1 mice at different ages prior and after the onset of the disease and age-matched control mice. ^*^*p* ≤ 0.05, ^**^*p* ≤ 0.01, ^***^*p* ≤ 0.001.

Supplementary Figure 6 Diseased-BWF1 mice present an increase in the frequency but not in the absolute number of regulatory T cells in the thymus. FACS analysis of regulatory T cells in BWF1 mice at different ages prior and after the onset of the disease and age-matched control mice. **(A)** Representative example of Foxp3 and CD25 expression in thymocytes CD4^+^SP (CD4+CD8–) from diseased-BWF1 and age-matched control mice. **(B)** Frequency (left) and absolute number (right) of regulatory T cells in BWF1 mice at different ages prior and after the onset of the disease and age-matched control mice. Student's *t*-test, ^*^*p* ≤ 0.05; ^***^*p* ≤ 0.001.

Supplementary figure 7 Thymic follicular helper T cells from diseased-BWF1 express Bcl-6 transcription factor. **(A)** Flow cytometry plots of PD-1^+^CXCR5^+^ T follicular helper cells (T_FH_) and Non-T_FH_ (PD-1^−^CXCR5^−^) of diseased-BWF1 mice (left). Analysis was carried out in a CD4SP (CD4^+^CD8^−^) gate. Analysis of Bcl-6 expression in T_FH_ and Non-T_FH_ from thymus of diseased-BWF1 mice (right). **(B)** Summary of Bcl-6 expression in two independent experiments. MFI, Median fluorescence intense.

Supplementary Figure 8 Thymic B cells from diseased-BWF1 and age-matched control mice express similar levels of co-stimulation and antigen presentation molecules. The expression of CD83, CD86, CD40, and I-Ad in thymic B cells of diseased-BWF1 and age-matched-control mice was assessed by FACS in a CD19^+^CD11c^−^ gate.

Supplementary Figure 9 Splenic B cells induce follicular helper T differentiation from thymocytes. Frequency of PD-1^+^CXCR5^+^ follicular helper T cells (in a CD4^+^CD8^−^ gate) 5 days after co-culture of thymocytes (from 3 m-control mice) with splenic B cells from diseased-BWF1 or age-matched control mice, in presence of IL-7 (6 ng/ml).

Supplementary Figure 10 Thymic B cells from diseased-BWF1 mice favor the expansion of follicular helper T cells in an OX40L-dependent manner. **(A)** Flow cytometry plots of PD-1^+^CXCR5^+^ follicular helper T cells (in a CD4^+^CD8^−^ gate) 5 days after co-culture. Thymocytes (from 3 m-control) were cultured with thymic B cells from diseased-BWF1 in presence of IL-7 (6 ng/mL) in all conditions and in the presence or absence of an αOX40L blocking antibody (clone RM134L, 10 μg/mL). **(B)** Proliferation of CD4^+^SP populations 5 days after co-culture with thymic B cells as assessed by cell trace violet dilution.

Supplementary Table 1 RNAseq. List of genes upregulated in thymic B cells from diseased-BWF1 compared to thymic B cells from age-matched control mice. The genes in the list were selected with at least 1.5-fold change and *p*-value < 0.05.

Supplementary Table 2 RNA-seq. Genes downregulated in thymic B cells from diseased-BWF1 compared to thymic B cells from age-matched control mice. The genes in the list were selected with at least 1.5-fold change and *p* value < 0.05.
