# Supplementary material for: Thymic B Cells Promote Germinal Center-Like Structures and the Expansion of Follicular Helper T Cells in Lupus-Prone Mice
Source: Front Immunol. 2020 Apr 28;11:696. doi: 10.3389/fimmu.2020.00696 (PMC7199236; doi:10.3389/fimmu.2020.00696)

Supplementary Figure 1

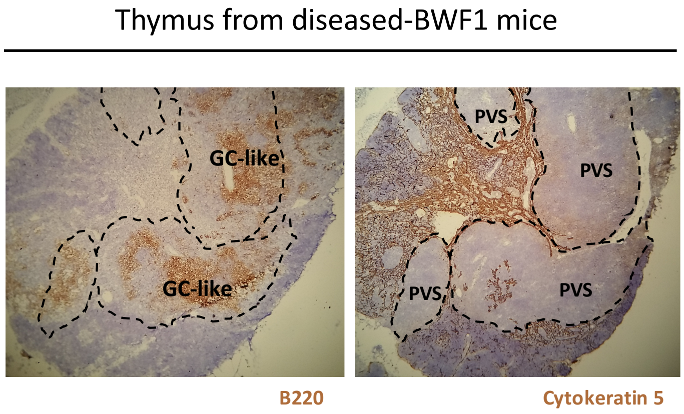

Supplementary Figure 2

A.

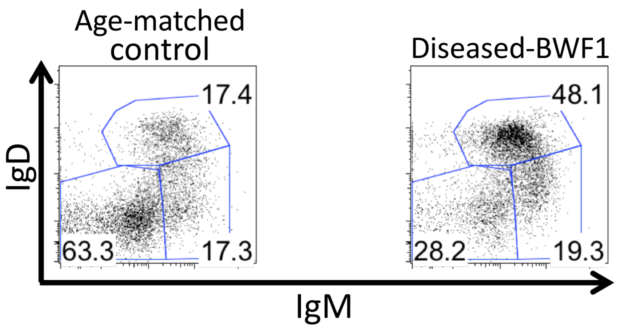

B.

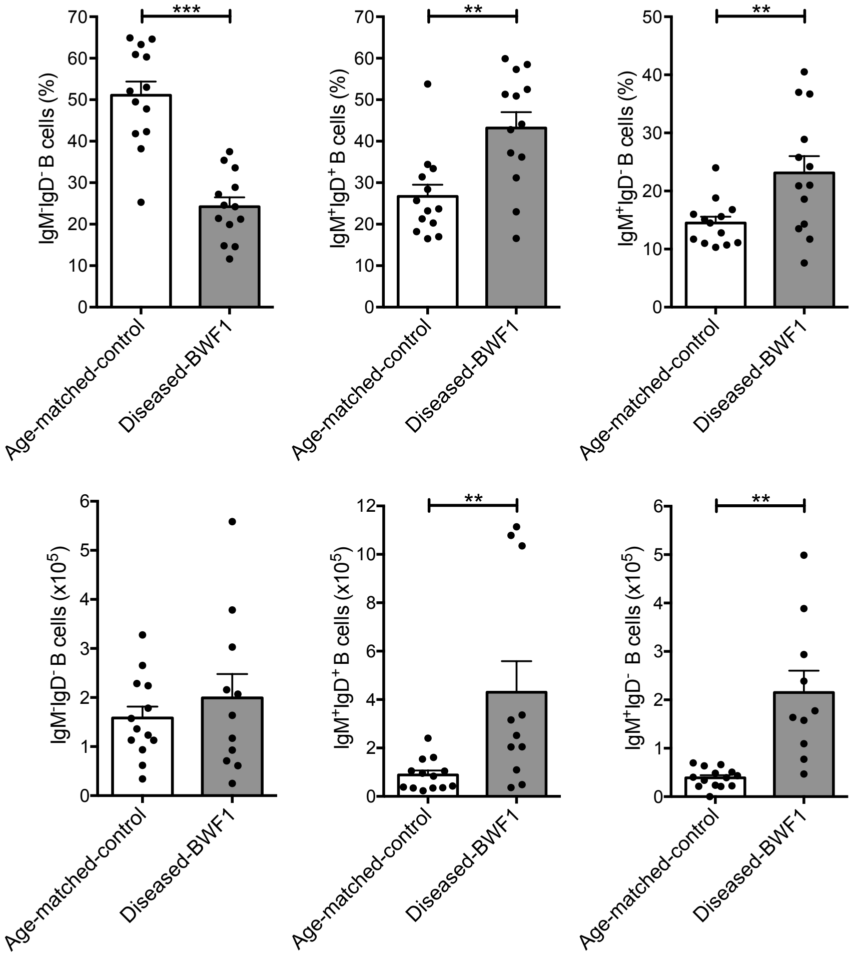

Supplementary Figure 3

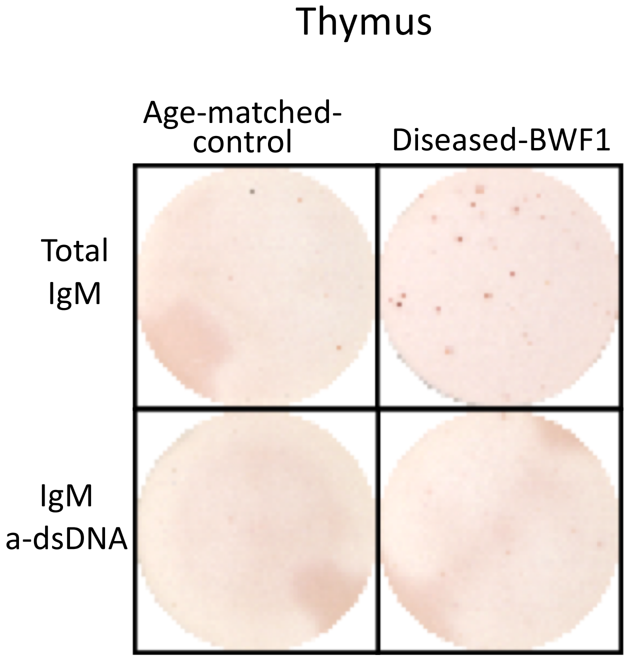

Supplementary Figure 4

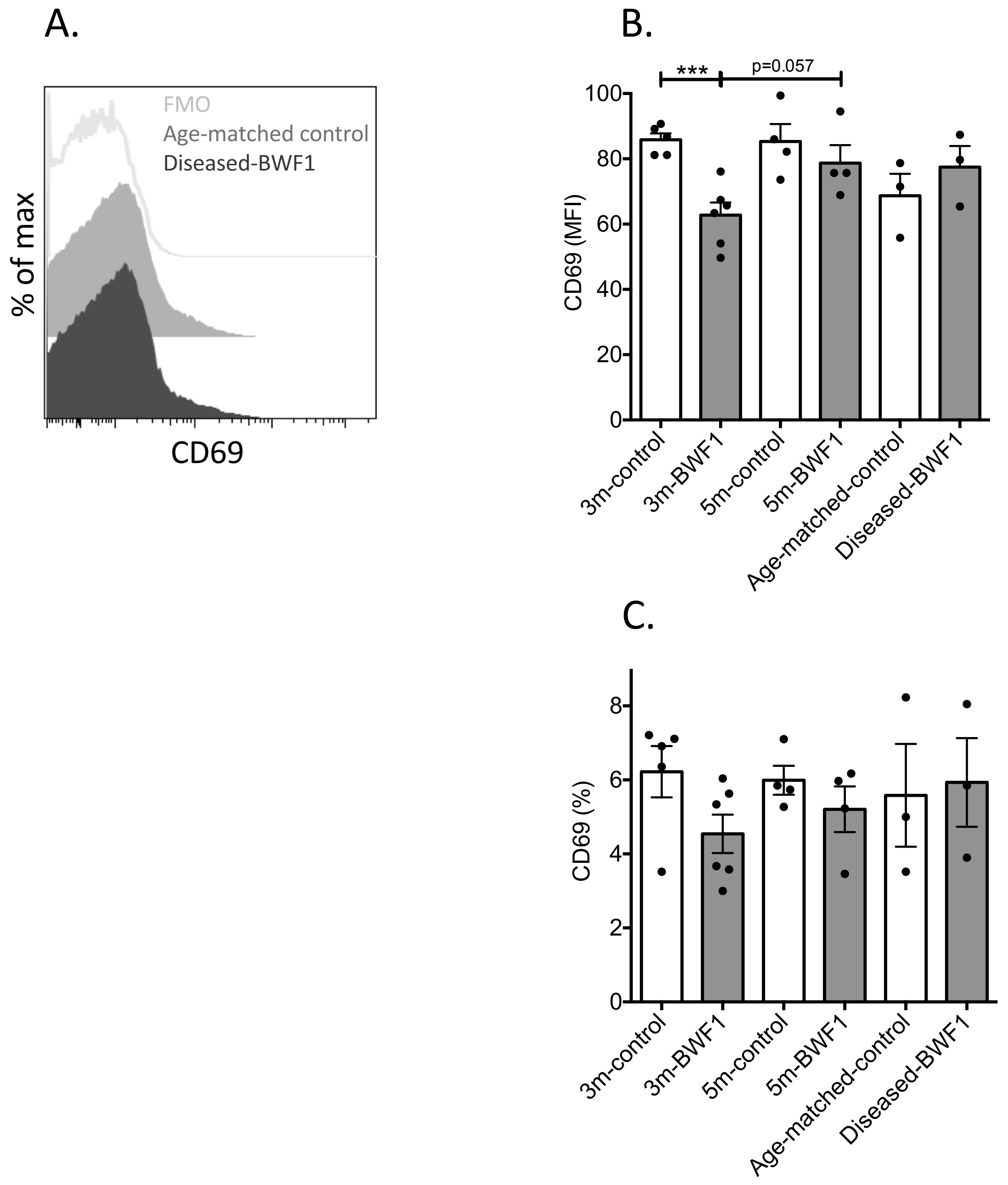

Supplementary Figure 5

A.

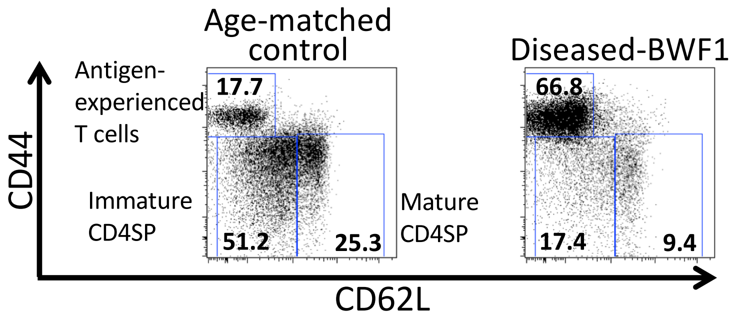

B.

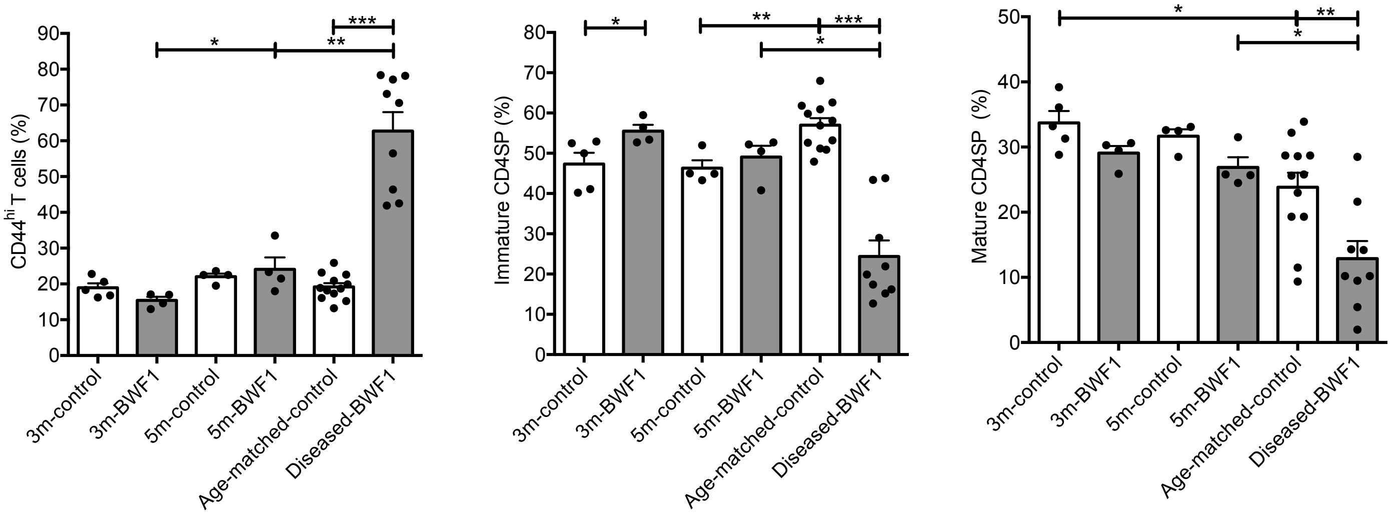

Supplementary Figure 6

A.

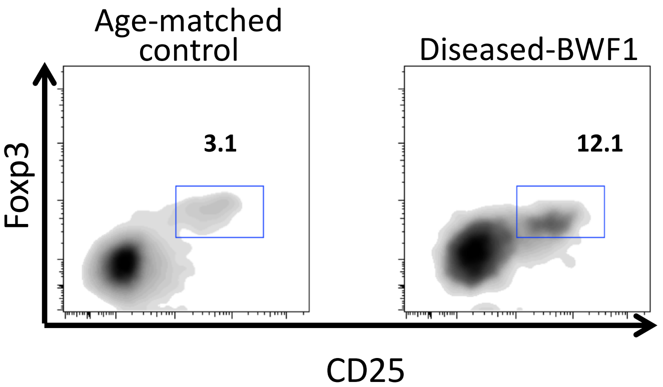

B.

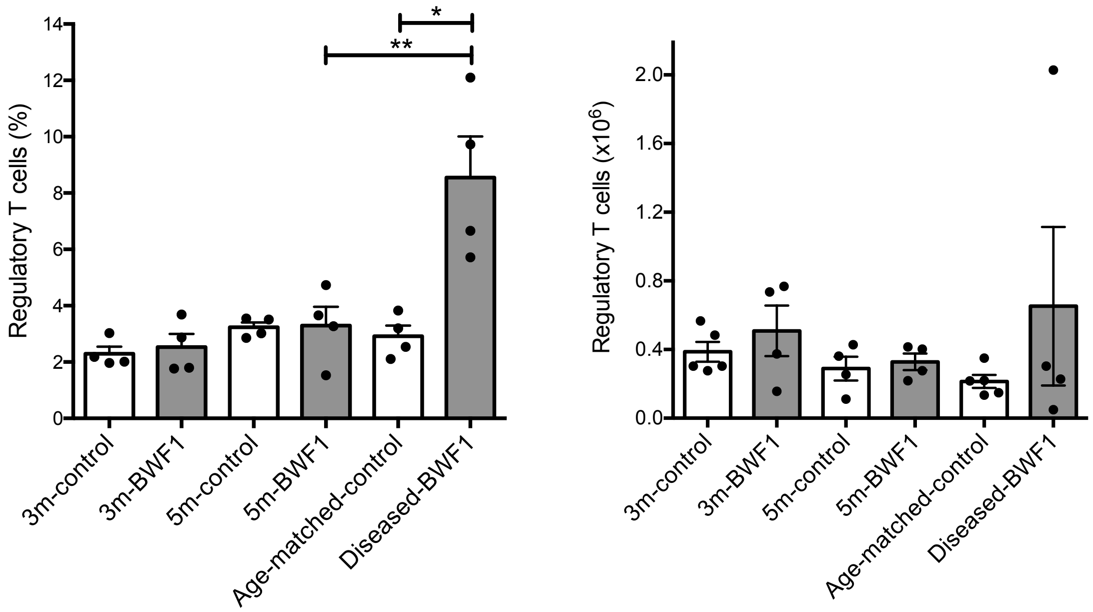

Supplementary Figure 7

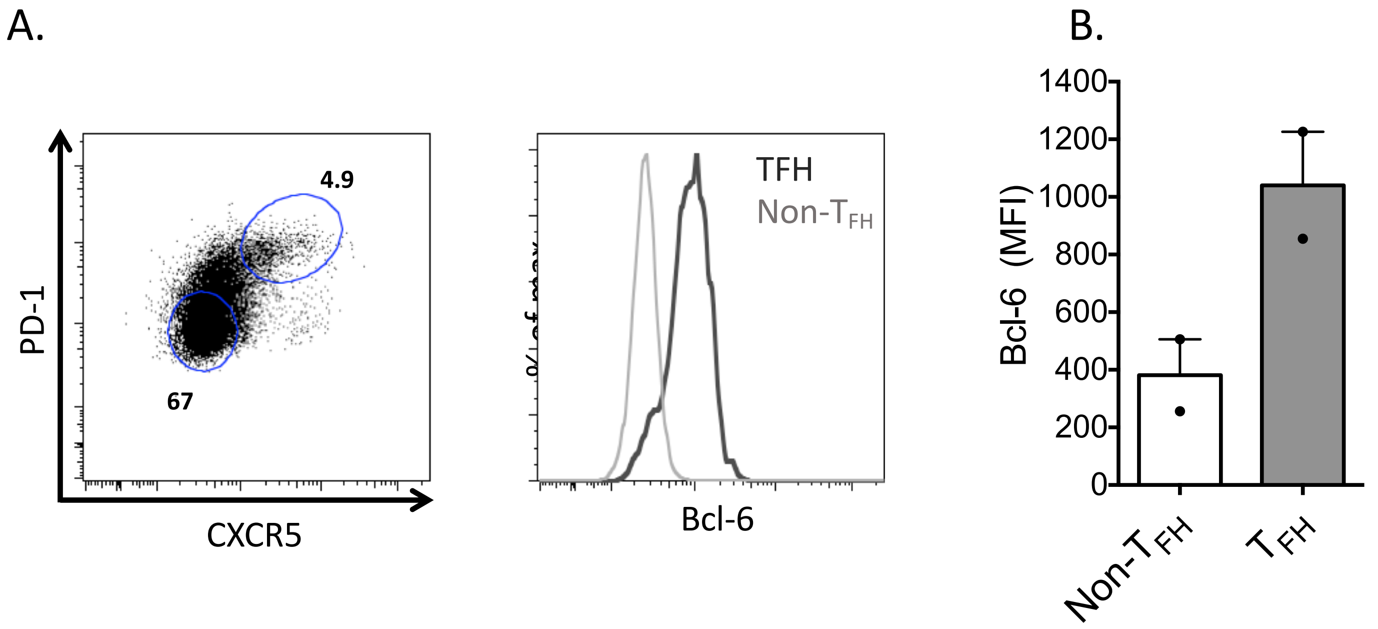

Supplementary Figure 8

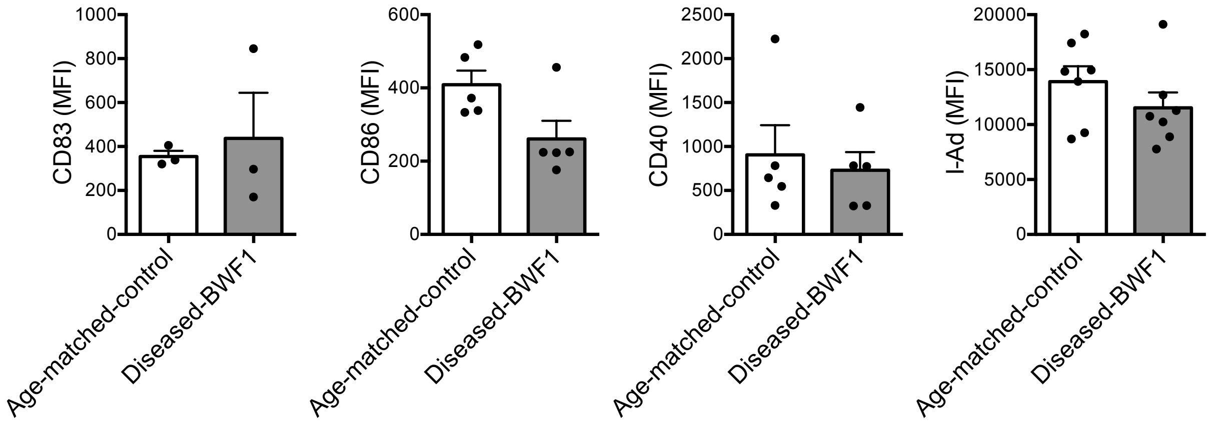

Supplementary Figure 9

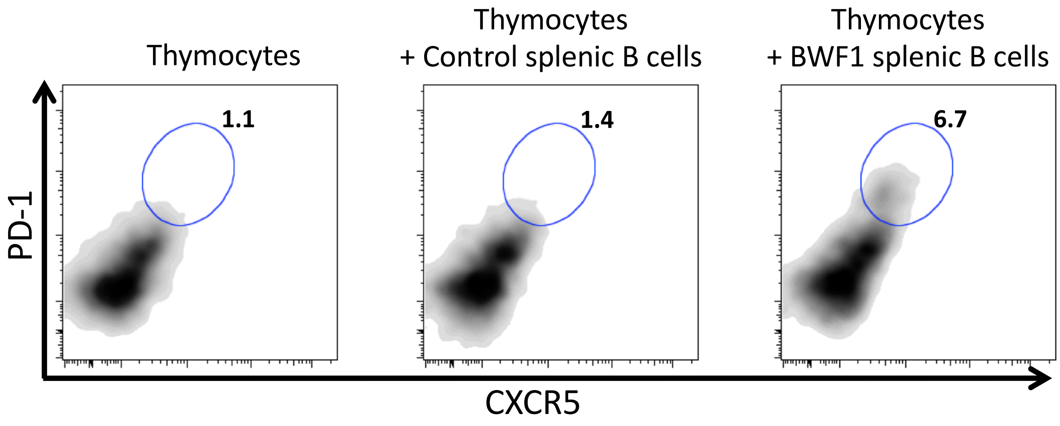

Supplementary Figure 10

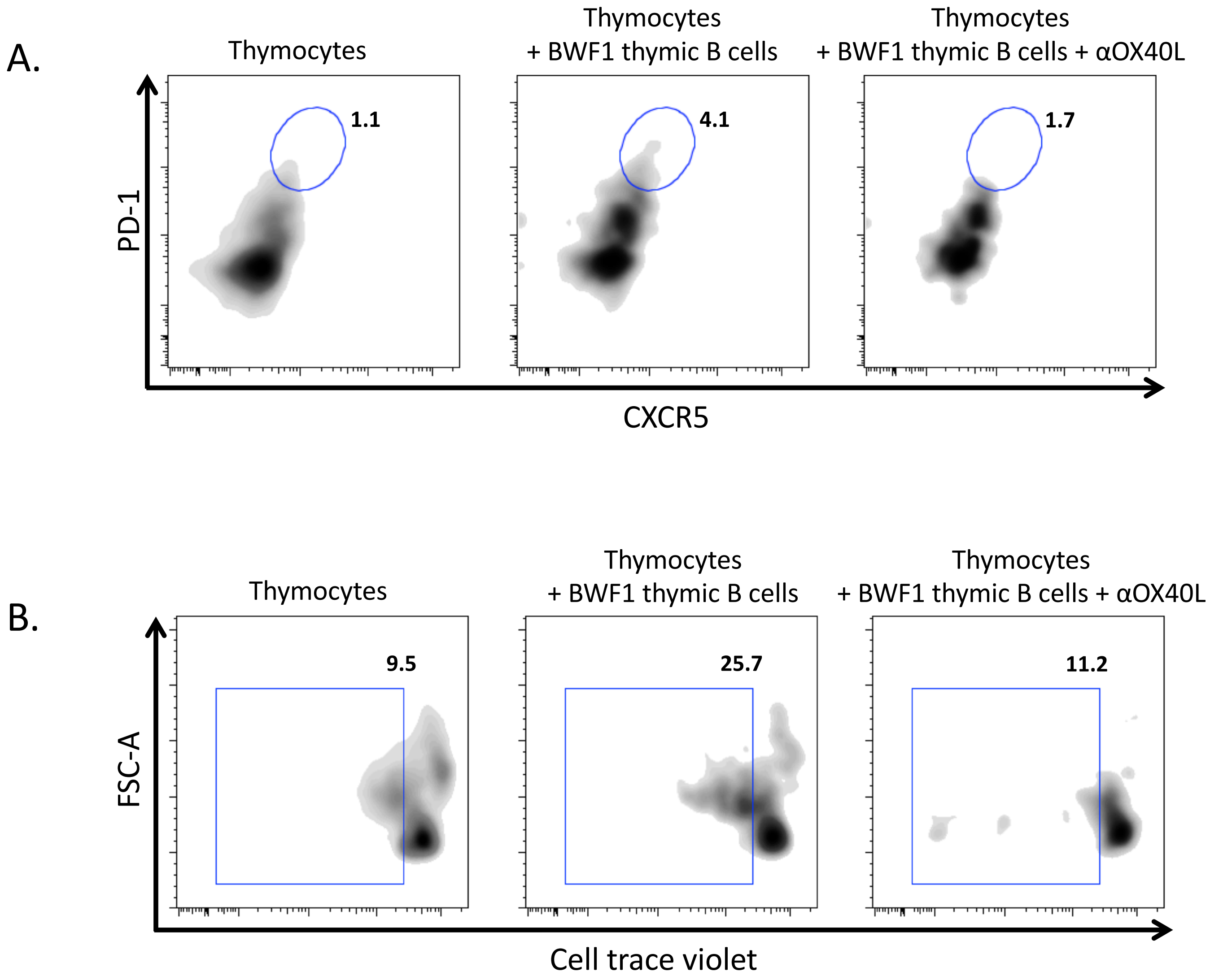

Supplement: Supplementary Figure 1 — Thymic B cells from diseased-BWF1 mice are localized in perivascular spaces. Representative images of thymic tissue from diseased-BWF1 stained with B220 (left panel) and cytokeratin 5 (right panel). [file Data_Sheet_1.pdf]
