# Supplementary material for: Thymic B Cells Promote Germinal Center-Like Structures and the Expansion of Follicular Helper T Cells in Lupus-Prone Mice
Source: Front Immunol. 2020 Apr 28;11:696. doi: 10.3389/fimmu.2020.00696 (PMC7199236; doi:10.3389/fimmu.2020.00696)
Supplement: Supplementary Table 1 — RNAseq. List of genes upregulated in thymic B cells from diseased-BWF1 compared to thymic B cells from age-matched control mice. The genes in the list were selected with at least 1.5-fold change and p-value < 0.05. [file Data_Sheet_2.docx]

**Supplementary table 1.** RNAseq. List of genes upregulated in thymic B cells from diseased-BWF1 compared to thymic B cells from age-matched control mice. The genes in the list were selected with at least 1.5-fold change and p-value < 0.05.

**Supplementary table 2.** RNA-seq. Genes downregulated in thymic B cells from diseased-BWF1 compared to thymic B cells from age-matched control mice. The genes in the list were selected with at least 1.5-fold change and p value < 0.05.
